# Pooled prevalence of psychological distress and mental health conditions in older adults with inflammatory bowel disease: a protocol for a systematic review and meta-analysis

**DOI:** 10.1186/s13643-026-03106-z

**Published:** 2026-02-20

**Authors:** Mark Momoh Koroma, Justin Chan, Mielle Michaux, Bwalya Kasanda, Kevan Jacobson

**Affiliations:** 1 Department of Pediatrics, Faculty of Medicine, University of British Columbia, Vancouver, BC Canada; 2 BC Children’s Hospital Research Institute, Vancouver, BC Canada; 3 College of Science and Engineering, University of Edinburgh, Edinburgh, United Kingdom

**Keywords:** Inflammatory Bowel Disease, Crohn’s disease, Ulcerative colitis, Older adults, Depression, Anxiety, Stress, Psychological distress, Mental health

## Abstract

**Background:**

Older adults (≥60 years) are one of the fastest-growing age groups, with a rising prevalence of chronic conditions such as inflammatory bowel disease (IBD). Mental health conditions are common among people living with IBD; however, the burden of depression, anxiety, and stress/psychological distress, specifically among older adults with IBD, remains poorly characterized. Most available evidence comes from younger or mixed-age cohorts, underscoring the need to synthesize evidence explicitly focused on older adults to address this gap.

**Methods/design:**

We will conduct a systematic review and, where applicable, a meta-analysis following PRISMA-P guidance. The protocol is registered in PROSPERO (ID: (CRD420251157744)). We will include quantitative observational studies (cross-sectional, cohort, and case-control) published in English-language journals from January 2014 to the present. Eligible studies must report extractable data for older adults (≥60 years, or ≥65 years where used) with IBD (Crohn’s disease, ulcerative colitis, or IBD-unclassified) and at least one eligible mental health outcome (depression, anxiety, and/or stress/psychological distress). Searches will be conducted in MEDLINE, EMBASE, PsycINFO, and CINAHL, and supplemented with reference-list screening, forward citation searching, and targeted grey literature searches using a pre-specified approach. Two reviewers will independently screen records, extract data, and assess risk of bias using design-appropriate tools. Pooled prevalence will be estimated using random-effects meta-analyses, with an appropriate transformation for proportions. Heterogeneity will be explored using I² and pre-specified subgroup/sensitivity analyses. Where pooling is not appropriate due to substantial heterogeneity, findings will be summarized using structured narrative synthesis. Where eligible longitudinal or comparative observational studies are available, association estimates (e.g., OR/RR/HR) will be synthesized and reported separately from prevalence outcomes.

**Discussion:**

This review may provide a consolidated synthesis of international evidence on the prevalence and pooled prevalence of depression, anxiety, and stress/psychological distress among older adults with IBD. Where data allow, it may also summarize association evidence as a separate stream. Findings may inform integrated care pathways for older adults with IBD, helping to identify key sources of heterogeneity and evidence gaps that can inform future research and care planning.

**Supplementary Information:**

The online version contains supplementary material available at 10.1186/s13643-026-03106-z.

## Background

The older population (≥60) is experiencing increased growth, driven by a decline in birth rates and an increase in life expectancy [1–3]. This demographic shift reflects a combination of factors, including higher educational attainment, increased access to family planning, advancements in socioeconomic and health sectors, which lead to a decrease in mortality [4–7]. Early in the 1980 s, the estimated number of individuals in this age group was around 250 million and was projected to reach approximately 760.7 million by 2025 [8]. However, this projection was exceeded, reaching 1 billion in 2020, and is estimated to increase to 1.4 billion by 2030 [9]. This age group often faces unique health challenges, such as multiple chronic health conditions, frailty, and declining immunity, which significantly affect their quality of life [10–12]. The complexity of chronic multi-morbid conditions usually makes treatment complicated, as many factors, such as drug interactions, are involved. Additionally, older adults are often underrepresented in research, frequently excluded from clinical trials due to comorbidities that could complicate treatment [13, 14]. At the molecular and cellular levels, older adults accumulate damage over time that gradually deteriorates their physical and mental abilities, serving as a hallmark of chronic conditions [15].

Inflammatory Bowel Disease is one of the chronic conditions increasingly diagnosed in older adults [16, 17]. It is a complex gastrointestinal disease with significant debilitating impacts [18, 19], characterized by various distressing symptoms such as persistent abdominal pain, chronic diarrhea, severe fatigue, and unintentional weight loss [20–22]. Consequently, IBD interferes with daily life and diminishes quality of life, which can be especially severe among older adults. Data from Ontario showed that the annual increase in IBD prevalence among older adults was estimated at 5.2%, higher than the 3.9% increase among the non-older adults during 1999-2008 [23]. In another study, the prevalence of IBD among older adults in Canada increased from 1 in 160 in 2018 to 1 in 88 in 2023 [24]. Recent research indicates a rise in IBD diagnoses among older adults and a second peak incidence [25–27]. Older age has been associated with physiological and psychological changes, including alterations in gut microbiota and a decline in cognitive function [28–30].

In fact, studies have shown a bidirectional relationship between the gut and the brain via multiple communication pathways [30–32]. These bidirectional communication pathways are disturbed during IBD and may influence a person's emotional and psychological balance [33, 34]. Moreover, mental health conditions such as anxiety and depression have been known to be associated with IBD and may be linked to worse IBD outcomes in the general population [35, 36]. It is estimated that about 14% of older adults (60 and above) have mental health disorders, with a quarter of them dying from suicide [37]. Older adults also experience significant cognitive impairment, which likely exacerbates mental health issues and worsens IBD outcomes in them.

This study addresses the lack of systematic reviews and meta-analyses focused specifically on the prevalence of mental health conditions among older adults with IBD. We plan to systematically review existing literature to determine how common these conditions are in this population, aiming to provide clear pooled prevalence estimates of depression, anxiety, and stress/psychological distress. Where eligible longitudinal or comparative observational studies are available, we will separately summarize association evidence (reported as a distinct evidence stream) rather than combining it with prevalence estimates in the same meta-analysis.

## Review objectives

### Primary objectives

The primary objective of this systematic review and meta-analysis is to estimate the prevalence and pooled prevalence of psychological distress and specified mental health conditions among older adults (aged ≥60 years) with IBD. Specifically, we aim to:

1. Estimate the prevalence and pooled prevalence of depression, anxiety, and stress/psychological distress (or closely related distress constructs) among older adults with IBD.
2. Estimate pooled prevalence stratified by IBD subtype (Crohn’s disease, ulcerative colitis, and IBD overall), were reported.
3. Compare prevalence patterns by sex (male vs female), where reported, and assess differences across IBD subtypes when data permit.
4. Compare prevalence estimates by mental health ascertainment method (e.g., clinician diagnosis/administrative codes vs structured diagnostic interviews vs validated instruments with stated thresholds).

### Secondary objectives

1. Associations between IBD and incident psychiatric disorders/mental health conditions (e.g., incident depression/anxiety).
2. Associations between baseline mental health conditions/psychological distress and subsequent IBD-related outcomes (e.g., disease activity/relapse/exacerbation, recurrence, hospitalization, surgery/stoma, and health-related quality of life), as defined by individual studies.
3. Identify evidence gaps in older-adult IBD and mental health research, including limitations in age-stratified reporting, outcome definitions, and availability of longitudinal data to inform future research priorities.

## Method/design

### Study method

This protocol outlines a systematic review study that may include a meta-analysis if sufficient data are available. The study will follow PROSPERO registration requirements and PRISMA guidelines checklist to achieve the objectives. Where applicable, prevalence estimates and association estimates will be synthesized as distinct evidence streams and will not be aggregated in the same meta-analysis.

### Study criteria

#### Inclusion and exclusion criteria

Studies will be selected based on pre-specified criteria related to the population of interest, study design, publication date, language of publication, and the outcome of interest.

##### Study types

This systematic review will include quantitative observational studies (cohort, cross-sectional, and case-control) that report eligible prevalence estimates and/or association effect measures for the older-adult subgroup.

Randomized clinical trials, intervention studies, qualitative studies, case reports/series, editorials, and reviews will be excluded. These designs do not provide appropriate prevalence estimates or comparable observational association measures for this review’s objectives.

##### Study population

We will include all studies of older adults diagnosed with IBD (Crohn's Disease, Ulcerative Colitis, or unspecified IBD) that report at least one mental health condition. Our primary operational definition of older adults is ≥60 years; studies defining older adults as ≥65 years will also be included, recorded as such, and considered in stratified analyses where feasible. Mixed-age studies will be included only if mental health outcomes are extractable for the older-adult subgroup, as applicable. In situations where the age stratification of older adults is limited to demographics and IBD, but not to mental health conditions, the authors of such studies will be contacted via the email address provided by the correspondent. If subgroup data remains unavailable after author contact and subsequent follow-up, the study will be documented and excluded from the primary prevalence synthesis due to non-extractable older-adult mental health outcomes.

##### Exposure

Inflammatory Bowel Diseases and mental health conditions/psychological distress will be defined using pre-specified ascertainment approaches. Inflammatory Bowel Diseases may be identified using:

1. Clinician diagnosis,
2. Established diagnostic criteria,
3. Administrative codes (ICD-9/ICD-10 or equivalent), or
4. Validated registry definitions.

Mental health conditions/psychological distress (e.g., depression, anxiety, stress/psychological distress) will be considered eligible when identified using any of the following pre-specified ascertainment approaches:

1. Clinician diagnosis and/or administrative diagnostic codes (ICD-9/ICD-10 or equivalent).
2. Structured diagnostic interviews (e.g., SCID, MINI, or comparable instruments); and/or
3. Validated screening instruments with clearly stated thresholds/cut-offs (e.g., GDS, PHQ-9, HADS, GAD-7, CES-D, K6/K10). We will not require ICD coding for eligibility.

#### Outcomes

Outcomes will be separately defined into primary and secondary outcomes.

Primary outcomes will focus on the prevalence and pooled prevalence of anxiety, depression, and stress or psychological distress among older persons with IBD. When data is available, we will record the number of older individuals with the condition and the total number of older adults with IBD. We will also note whether prevalence is point or period prevalence, as defined by each study.

Secondary outcomes will focus on association and will be analyzed as a separate stream. Suitable studies for association will include IBD-related measures linked to baseline mental health or psychological distress. These outcomes may include disease activity, relapses, recurrence, hospitalization, surgery, stoma formation, and health-related quality of life, as defined by each study. Where available, we will extract both adjusted and unadjusted association effect measures such as OR, RR, HR, or β coefficients, along with factors used in adjusted models. We will aggregate these association effect measures separately from prevalence estimates. If data are insufficient or too heterogeneous for meta-analysis in either stream, the findings will be summarized narratively using structured tables and ranges of estimates.

##### Time frame

This systematic review will include studies from January 2014 to the present. This range will be selected to ensure that the evidence is contemporary with current clinical practices, diagnostic criteria, and methodological standards. Within the past decade, there has been an increase in research characterizing older populations with IBD and co-morbid mental health conditions, largely facilitated by advances in electronic health records, administrative databases, and standardized diagnostic classifications. Focusing the review on this period will help detect current epidemiological trends, reflect recent changes in health system structure and population characteristics, and reduce heterogeneity caused by outdated definitions or methodologies in older studies.

##### Language

We will include only studies published in English, as it is less feasible to translate non-English studies into the language used by the review team. Finally, most high-quality, relevant biomedical and epidemiological literature on inflammatory bowel disease and mental health is published in English, suggesting comprehensive coverage of the evidence. For the sake of transparency, we will tabulate and report the non-English full-text articles that we identified and excluded during the full-text review stage. Reasons for exclusion will be captured in the PRISMA flow diagram and characterized as a potential biases in the limitations section.

##### Data sources

The following electronic databases will be searched: Medline/PubMed and EMBASE via Ovid, APA PsycINFO and CINAHL, EBSCOhost, and Scopus. We will also perform additional research to find eligible studies not captured by the selected database indexing. This will consist of (i) targeted grey literature searches based upon specified sources (to be detailed in the final search report) and (ii) backward and forward citation chasing (snowballing) of all included studies and appropriate reviews. We will document and report all additional sources searched, the dates of searches, and the total number of records retrieved per source.

### Search strategy

The search strategies will use a combination of controlled vocabulary (e.g., MeSH in MEDLINE, Emtree in Embase, CINAHL Subject Headings, PsycInfo Thesaurus) and free-text keywords to describe three major concepts:

- Inflammatory Bowel Disease (e.g., Crohn's disease, ulcerative colitis, IBD).
- Aged (e.g., older adults, elderly,, geriatric, older adults, aged 60+ years, aged 65+ years); and
- Mental Health and Psychosocial Distress (e.g., mental health, psychological distress, depression, anxiety, psychosocial factors).

#### Search strategy development and peer review

An experienced librarian of health sciences will guide the development of the search strategies. This will then be peer-reviewed using the Peer Review of Electronic Search Strategies (PRESS) checklist to ensure accuracy and comprehensiveness. Search sensitivity and specificity will be optimized by using Boolean operators (AND, OR) and truncation. Filters will be applied to restrict the search to humans and English-language articles. However, we will not apply database age-limit filters since older-adult subgroup results are not consistently indexed. Instead, we will incorporate age-related free-text terms such as older adult, elderly, senior, geriatric, and the aged. We will screen for studies reporting extractable data on older adults, even when records are not indexed under older-adult headings. The complete MEDLINE search strategy will be provided in an appendix and adapted for the other databases, with final search dates reported in a supplementary file. Reference list screening and forward citation searching of included studies will be conducted to identify additional eligible studies. Records will be managed in Covidence.

##### Screening process

Two reviewers will independently evaluate titles and abstracts for eligibility using a screening checklist (Table 1). Complete texts of potentially eligible articles will be obtained and evaluated against the eligibility requirements. Discrepancies will be addressed through dialogue or a third party. Before the formal screening, reviewers will do a pilot calibration exercise on a sample of records to enhance and standardize the application of eligibility criteria. Inter-rater agreement will be evaluated (e.g., utilizing percent agreement and/or Cohen’s kappa) during the pilot phase and monitored as necessary. All screening processes will be conducted in Covidence, with justifications for full-text exclusion documented.

**Table 1:**
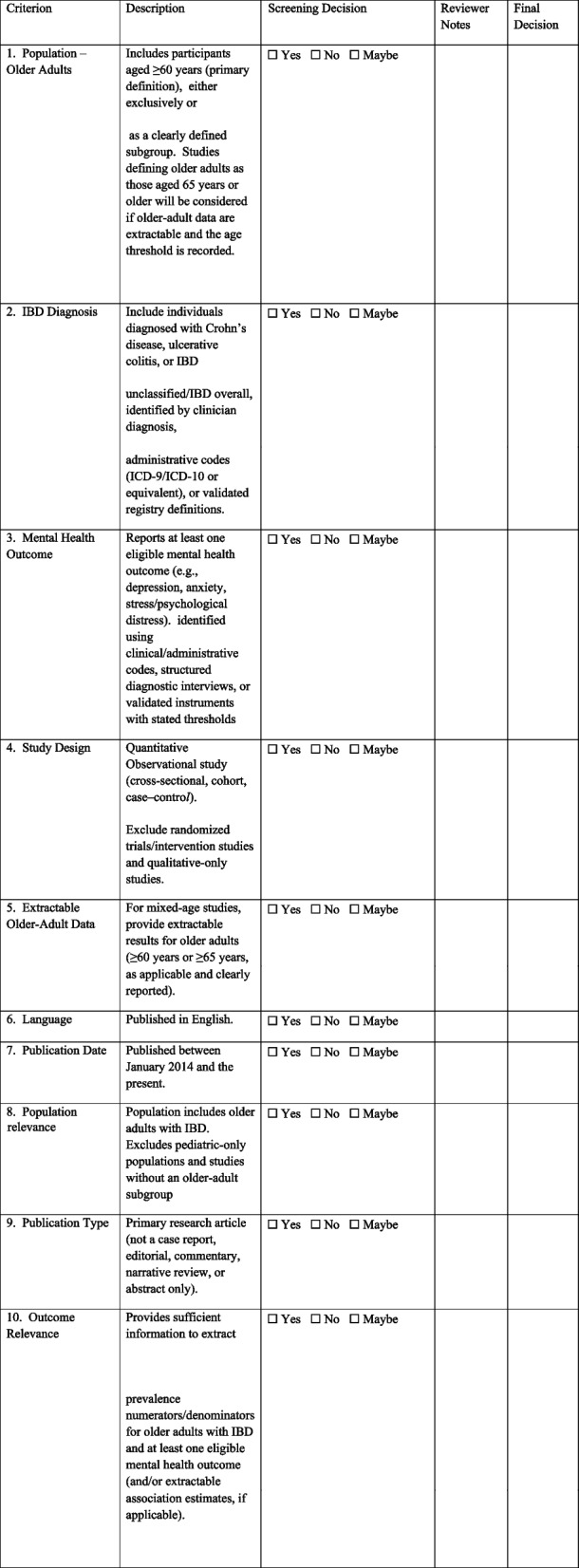
Reviewers screening checklist (Title/abstract and full-text screening)

#### Data extraction

Data extraction will be piloted using a designated Excel form on a subset of the included studies (approximately 5–10 studies, or approximately 10% of the included studies, whichever is smaller). Two reviewers will independently extract data during this pilot phase to calibrate the extraction fields and ensure consistent interpretation of variables. Discrepancies will be resolved through discussion or third-party adjudication. Following calibration, the finalized extraction form will be implemented in Covidence for full data extraction across all included studies. Table 2 will serve as a data-extraction template for all included studies in the prevalence stream while Table 3 will be used to extract data from the association stream. Extracted data will include study identifiers, setting, design, sample size, IBD definition, mental health ascertainment method and thresholds, age definition for the older-adult subgroup, numerator/denominator for prevalence estimates, and (where applicable) association effect estimates and covariates. All extracted data will be retained as a finalized, reconciled dataset for analysis, with changes documented through version control. Where both reviewers extract data from the same study, any differences will be reconciled, and a final, agreed-upon dataset will be retained for analysis.

**Table 2:** Data extraction template for included studies (Prevalence evidence stream)

**Data extraction field**	**Extracted data/notes**
**I. Study identification and context**	
Study ID	
First author (Year)	
Study Title	
Journal	
Country/region	
Setting (e.g., community, outpatient, inpatient)	
Data Source (e.g., registry, administrative claims, EHR, clinic sample)	
Funding/conflicts noted.	
**II. Study design and methodology**	
Study Design (cross-sectional/cohort/case-control)	
Recruitment Period (Start-End)	
Eligibility Criteria (as reported)	
Older-adult definition used in study (e.g., ≥60, ≥65)	
IBD subgroup included (CD/UC/IBD)	
Sample size used for older-adult IBD analyses (N)	
Age summary for older-adult subgroup (mean±SD or median [IQR])	
Sex distribution for older-adult subgroup (%)	
**III. IBD and mental health ascertainment**	
IBD ascertainment method (clinical/criteria/codes/registry)	
Mental health ascertainment method (codes/structured interview/validated instrument)	
Instrument/interview name (if applicable)	
Threshold/cut-off used (if applicable)	
Diagnostic codes used for IBD and/or mental health (if reported)	
Psychological distress construct (depression/anxiety/stress/mixed; as defined)	
Prevalence type and time window (point/period/lifetime; timeframe as defined)	
**IV. Prevalence data (primary outcomes)**	
Mental health outcomes extracted (depression/anxiety/stress or psychological distress)	
Numerator(s): number for each mental health outcome	
Denominator(s): total older adults with IBD for each estimate	
Prevalence estimate(s) reported (%) (or computed from numerator/denominator)	
Stratified prevalence estimates (if reported) (e.g., by IBD subtype, sex, ascertainment method)	Record numerators/denominators for each stratum (e.g., IBD subtype, sex, ascertainment method). Each stratified estimate should also be entered as a separate row in the stratified prevalence table (Supplementary Table S2b)
Number of prevalence estimates extracted from the study	Count each distinct estimate (e.g., by subtype/sex/method) for transparent synthesis
**V. Association evidence stream (secondary; analyzed separately)**	
Eligible for association stream (Yes/No); reason if No	
Association exposure definition (e.g., baseline mental health or IBD status)	
Association outcome definition (incident psychiatric disorder or IBD-related outcome)	
Effect estimate(s) reported (OR/RR/HR/β) with 95% CI; adjusted vs unadjusted	
Covariates adjusted for (if applicable)	
Follow-up duration/time window (if applicable)	
**VI. Notes for synthesis**	
Key findings (brief)	
Notes/limitations reported by authors	
Duplicate/overlapping population check (notes)	
VI. Notes for synthesis	
Key findings (brief)

**Table 3:** Data extraction template for association evidence stream (Secondary). Association evidence will be synthesized separately from prevalence outcomes and will not be combined in the same meta-analysis. Complete this table only for studies eligible for the association evidence stream (longitudinal/cohort, case-control, or comparative observational designs reporting effect estimates)

**Data extraction field**	**Extracted data/notes**
**I. Study identification and context**	
Study ID	
First author (Year)	
Study Title	
Journal	
Country/region	
Setting (e.g., community, outpatient, inpatient)	
Data Source (e.g., registry, administrative claims, EHR, clinic sample)	
Funding/conflicts noted.	
**II. Study design and methodology**	
Study design (cohort/case-control/other comparative observational)	
Recruitment/Study period (Start–End)	
Eligibility criteria (as reported)	
Older-adult definition used in study (e.g., ≥60, ≥65)	
Older-adult subgroup inclusion method (age-stratified vs older-only cohort)	
Analysis sample size (older-adult subgroup N used in effect estimate)	
IBD subtype(s) included (CD/UC/IBD overall/IBD-U)	
**III. Association structure and directionality**	
Eligible for the association stream? (Yes/No)	
If Yes—evidence type	☐ IBD → incident MH outcome ☐ MH exposure → IBD outcome ☐ Other: ___
Directionality statement (e.g., Depression → hospitalization; IBD → incident depression)	
Comparator/reference group (if applicable)	
**IV. Exposure details (baseline)**	
Exposure variable (what exactly is the exposure?)	
Exposure ascertainment method	☐ ICD/administrative codes ☐ clinician diagnosis ☐ structured interview ☐ validated instrument ☐ other: ___
Instrument/code list and threshold/cut-off (if applicable)	
Timing of exposure assessment (baseline window)	
**V. Outcome details**	
Outcome variable (what exactly is the outcome?)	
Outcome definition (as reported)	
Outcome ascertainment method	☐ ICD/administrative codes ☐ clinician diagnosis ☐ registry ☐ patient-reported ☐ other: ___
Timing of outcome assessment	
**VI. Follow-up/time window**	
Follow-up duration (mean/median; range)	
Time window used (e.g., 1-year risk; 5-year incidence)	
Censoring rules/loss-to-follow-up handling (if reported)	
**VII. Effect estimates and statistical details**	
Effect measure reported	☐ HR ☐ OR ☐ RR ☐ β ☐ other: ___
Effect estimate + 95% CI (extracted)	
The extracted estimate is	☐ most adjusted ☐ minimally adjusted ☐ unadjusted
Statistical model (e.g., Cox, logistic, Poisson, linear, mixed)	
Confounding control approach	☐ multivariable regression ☐ matching ☐ propensity score ☐ weighting ☐ stratification ☐ other: ___
Covariates adjusted for (list key ones)	
Alternative models reported (brief)	
**VIII. Results summary and interpretation**	
Key finding (1–2 lines; plain language)	
Authors’ conclusion re association	
Mechanisms/pathways discussed (optional; brief)	
Notes/limitations (including reviewer notes)	

#### Handling of missing or unclear data

Where data are missing, ambiguous, or not stratified by age, corresponding authors will be contacted (three attempts within 2–4 weeks). To avoid excluding studies solely because authors do not respond, we will apply pre-specified decision rules: studies will be included in quantitative synthesis only when older-adult mental health outcomes are extractable from the publication or supplementary materials. When subgroup outcomes are not extractable, studies will be documented and summarized narratively as evidence gaps but excluded from quantitative synthesis. All author-contact attempts and final inclusion decisions will be recorded.

#### Quality assessment

The risk of bias will be assessed using validated tools appropriate to each study design (Supplementary Table S1a–S1c): the Newcastle–Ottawa Scale (NOS) for cohort and case-control studies, and the Joanna Briggs Institute (JBI) Critical Appraisal Checklist for Analytical Cross-Sectional Studies. Risk-of-bias assessments will be conducted independently by two reviewers, with disagreements resolved by consensus or adjudication by a third reviewer. Prior to full assessment, reviewers will pilot the risk-of-bias tools on a subset of included studies to calibrate their interpretation of the criteria. Inter-rater agreement will be assessed during this pilot phase using percent agreement and Cohen’s kappa to identify and resolve systematic discrepancies. Discrepancies will be discussed to harmonize interpretation. Risk-of-bias judgments will be incorporated into the interpretation of findings and into the GRADE certainty assessments for the primary prevalence outcomes, including pre-specified sensitivity analyses excluding studies rated as high risk of bias, and narrative discussion where quantitative synthesis is not appropriate.

#### Data analysis and synthesis plan

The extracted data will be analyzed and synthesized using narrative and quantitative approaches. Quantitative evidence will focus on prevalence and association analyses and will be reported as separate evidence streams. Study characteristics and outcomes will be summarized using descriptive statistics. Supplementary Table S2a will include summaries of the number of older adults with IBD and mental health conditions (e.g., depression/anxiety or stress). Supplemental Table S2b will present stratified summaries by mental health condition, IBD subtype, sex, and other relevant study-level variables, as reported. Where meta-analysis is not appropriate due to substantial clinical or methodological heterogeneity, findings will be summarized using structured narrative synthesis.

Prevalence meta-analysis: Where sufficient data are available, pooled prevalence estimates will be calculated using random-effects meta-analysis. Study-specific prevalence will be calculated as the proportion of older adults with IBD who meet the criteria for each mental health outcome (numerator: number with the condition; denominator: total older adults with IBD). We will record whether each estimate represents point or period prevalence, as defined by the study. We will pool proportions using a random-effects model with a logit transformation and will back-transform pooled estimates for reporting. Between-study variance (τ2) will be estimated using restricted maximum likelihood (REML). We will report pooled prevalence with 95% confidence intervals, I², τ2, and prediction intervals where appropriate.

##### Zero events and extreme proportions

Studies reporting zero events or 100% prevalence will be retained. Where required by the logit model, we will apply a small continuity correction and will report the exact correction used. As a pre-specified sensitivity analysis, we will repeat the prevalence meta-analysis using the Freeman–Tukey double arcsine transformation to assess robustness to zero-event and extreme-proportion studies.

##### Exploring heterogeneity

Pre-specified subgroup analyses will be conducted where sufficient studies are available, stratified by IBD subtype (Crohn’s disease, ulcerative colitis, and IBD overall), sex, and mental health ascertainment method (clinical/administrative codes, structured diagnostic interviews, and validated instruments with stated thresholds). Where relevant, we will also stratify by study design (cross-sectional vs cohort/case-control). Sensitivity analyses will include excluding studies at high risk of bias and evaluating the impact of key analytic decisions (transformation choice and continuity correction).

##### Meta-regression

Meta-regression will be conducted only when at least 10 studies are available for a given outcome. Pre-specified covariates will include mental health ascertainment method, setting/region, study design, and mean/median age of the older-adult subgroup, where reported. Meta-regression results will be interpreted cautiously.

##### Small-study effects and reporting bias

Small-study effects and reporting bias: When ≥10 studies contribute to a prevalence meta-analysis, we will explore small-study effects using funnel plots and regression-based tests for funnel plot asymmetry. Results will be interpreted cautiously because these approaches can be unreliable in proportion meta-analyses, particularly with extreme prevalence values and substantial heterogeneity. When fewer than 10 studies are available, we will describe potential reporting bias qualitatively (e.g., by comparing published estimates across settings and study sizes and noting evidence gaps).

##### Certainty of evidence

We will assess the certainty of evidence for the primary prevalence outcomes using an adaptation of the GRADE framework for prevalence (risk of bias, inconsistency, indirectness, imprecision, and publication bias). We will present certainty ratings in a “Summary of Findings”.

##### Association evidence stream (reported separately)

Where eligible longitudinal or comparative observational studies are available, association effect estimates (OR, RR, HR, or β coefficients) will be extracted and synthesized separately (Supplementary Table S1A-S1C) from prevalence estimates and will not be combined with prevalence outcomes in the same meta-analysis. Where pooling is appropriate and measures are sufficiently comparable, random-effects models will be used; otherwise, results will be summarized narratively.

## Discussion

The rapidly aging global population and the increasing prevalence of IBD and mental health conditions pose a significant clinical challenge in managing these conditions among older adults (≥60 years). While accumulating evidence exhibits mediating effects between mental health conditions (such as anxiety, depression, stress, and others) and IBD outcomes, older adults remain underrepresented in this domain. They are often not reported as a distinct age-stratified subgroup. The results of this systematic review and meta-analysis are anticipated to provide consolidated prevalence, and pooled prevalence estimates of key mental health conditions in older adults with IBD, and to summarize (as a separate evidence stream) observational evidence on associations between baseline mental health conditions/psychological distress and subsequent IBD-related outcomes where available. Critically, the findings may help inform clinicians and healthcare systems about the potential value of routine mental health screening and integrated care pathways tailored to older adults with IBD. Moreover, this review may help guide future research and policy by highlighting gaps in evidence specific to older adults, heterogeneity in measurement and ascertainment methods, and limitations in age-stratified reporting.

### Strengths and limitations

Differences in diagnostic criteria and mental health assessment tools across studies, as well as variations in healthcare systems, may limit this review and may contribute to substantial heterogeneity in prevalence estimates Furthermore, most mental health conditions may be underdiagnosed in older adults because of stigma or atypical presentations which may lead to underestimation of true prevalence As a result, quantitative pooling may not be appropriate for all outcomes or subgroups, and some findings may need to be summarized narratively. Key strengths include a librarian-supported search strategy, duplicate screening and extraction, and pre-specified subgroup analyses (e.g., by IBD subtype and mental health ascertainment method) to explore sources of heterogeneity. Limiting inclusion to English-language studies could also induce language bias; however, much of the high-quality research in this domain is published in English. This study will be, to our knowledge, among the first systematic reviews and meta-analyses to compile worldwide evidence on the prevalence of psychological distress and mental health conditions among seniors with IBD.

#### Dissemination plan

Findings will be submitted for publication in a peer-reviewed journal and presented at relevant academic conferences. The final review will be reported in accordance with the PRISMA 2020 guidelines, and key supplementary materials (e.g., search strategies and extraction templates) will be provided as appendices or supplementary files, where feasible.

#### Registration

This protocol was registered in PROSPERO (CRD420251157744). Any protocol amendments will be documented and updated in PROSPERO.

## Supplementary Information

Supplementary Material 1

Supplementary Material 2

## References

[1] Brunborg H. Increasing life expectancy and the growing elderly population. Nor Epidemiol. 2012;22(2):75–83. 10.5324/nje.v22i2.1552.

[2] Shrestha LBJHa. Population aging in developing countries: the elderly populations of developing countries are now growing more rapidly than those in industrialized nations, thanks to health advances and declining fertility rates. Health Aff. 2000;19(3):204–12. 10.1377/hlthaff.19.3.204 10812800

[3] Mathers CD, et al. Causes of international increases in older age life expectancy. Lancet. 2015;385(9967):540–8. 25468166 10.1016/S0140-6736(14)60569-9

[4] Aitken RJ. The changing tide of human fertility. Hum Reprod. 2022;37(4):629–38. 35079808 10.1093/humrep/deac011 PMC8977063

[5] Liu DH, Raftery AE. How do education and family planning accelerate fertility decline? Popul Dev Rev. 2020;46(3):409–41. 33132461 10.1111/padr.12347 PMC7590131

[6] Miladinov G. Socioeconomic development and life expectancy relationship: evidence from the EU accession candidate countries. Genus. 2020;76(1):2.

[7] Balaj M, et al. Effects of education on adult mortality: a global systematic review and meta-analysis. Lancet Public Health. 2024;9(3):e155–65. 38278172 10.1016/S2468-2667(23)00306-7 PMC10901745

[8] Hauser PM. Aging and increasing longevity of world population. In: Häfner H, Moschel G, Sartorius N, editors. Mental health in the elderly: a review of the present state of research. Berlin, Heidelberg: Springer; 1986. p. 9–14. 10.1007/978-3-642-70958-6_2.

[9] World Health Organization. Ageing and Health. 2025. Available from: https://www.who.int/news-room/fact-sheets/detail/ageing-and-health. Cited 13 October 2025.

[10] Boah M, et al. Frailty, multimorbidity and quality of life in an ageing population in Africa: a cross-sectional, population-based study in rural and urban Rwanda. Fam Med Community Health. 2025;13(4):e003512. 41052895 10.1136/fmch-2025-003512 PMC12506473

[11] Maresova P, et al. Consequences of chronic diseases and other limitations associated with old age – a scoping review. BMC Public Health. 2019;19(1):1431. 31675997 10.1186/s12889-019-7762-5 PMC6823935

[12] Kim DH, Rockwood K. Frailty in older adults. N Engl J Med. 2024;391(6):538–48. 39115063 10.1056/NEJMra2301292 PMC11634188

[13] Kochar B, et al. Systematic review of inclusion and analysis of older adults in randomized controlled trials of medications used to treat inflammatory bowel diseases. Inflamm Bowel Dis. 2021;27(9):1541–3. 33705536 10.1093/ibd/izab052

[14] van Marum RJ. Underrepresentation of the elderly in clinical trials, time for action. Br J Clin Pharmacol. 2020;86(10):2014–6. 32909294 10.1111/bcp.14539 PMC7495271

[15] Aunan JR, et al. Molecular and biological hallmarks of ageing. Br J Surg. 2016;103(2):e29-46. 26771470 10.1002/bjs.10053

[16] Danpanichkul P, et al. Global epidemiology and burden of elderly-onset inflammatory bowel disease: a decade in review. J Clin Med. 2023. 10.3390/jcm12155142. 37568544 10.3390/jcm12155142 PMC10420121

[17] Taleban S, et al. Inflammatory bowel disease and the elderly: a review. J Crohns Colitis. 2015;9(6):507–15. 25870198 10.1093/ecco-jcc/jjv059

[18] Kemp K, Griffiths J, Lovell K. Understanding the health and social care needs of people living with IBD: a meta-synthesis of the evidence. World J Gastroenterol. 2012;18(43):6240–9. 23180944 10.3748/wjg.v18.i43.6240 PMC3501772

[19] Hall NJ, et al. The fight for “health-related normality”: a qualitative study of the experiences of individuals living with established inflammatory bowel disease (ibd). J Health Psychol. 2005;10(3):443–55. 15857873 10.1177/1359105305051433

[20] Singh S, et al. Common symptoms and stressors among individuals with inflammatory bowel diseases. Clin Gastroenterol Hepatol. 2011;9(9):769–75. 21645640 10.1016/j.cgh.2011.05.016

[21] Perler BK, et al. Correction to: Presenting symptoms in inflammatory bowel disease: descriptive analysis of a community-based inception cohort. BMC Gastroenterol. 2020;20(1):406. 33272202 10.1186/s12876-020-01526-2 PMC7713155

[22] Lennard-Jones JE. Classification of inflammatory bowel disease. Scand J Gastroenterol Suppl. 1989;170:2-6. discussion 16-9. 10.3109/00365528909091339 2617184

[23] Canada CsC. Impact of Inflammatory Bowel Disease. 2018. Crohn’s & Colitis Canada: Canada. p. Impact of Inflammatory Bowel Disease.

[24] Windsor JW, et al. The 2023 Impact of Inflammatory Bowel Disease in Canada: Executive Summary. J Can Assoc Gastroenterol. 2023; 6(Supplement_2):S1-S8. 10.1093/jcag/gwad003 PMC10478799 37674500

[25] Everhov ÅH, et al. Incidence and treatment of patients diagnosed with inflammatory bowel diseases at 60 years or older in Sweden. Gastroenterology. 2018;154(3):518-528.e15. 29102619 10.1053/j.gastro.2017.10.034

[26] Liu Y, et al. Global burden of inflammatory bowel disease in the elderly: trends from 1990 to 2021 and projections to 2051. 2024;5:2024. 10.3389/fragi.2024.1479928 PMC11540814 39512627

[27] Talks C. Managing Inflammatory Bowel Disease (IBD) in the Elderly. Canadian Digestive Health Foundation, 2022.

[28] Alsegiani AS, Shah ZA. The influence of gut microbiota alteration on age-related neuroinflammation and cognitive decline. Neural Regen Res. 2022;17(11):2407–12. 35535879 10.4103/1673-5374.335837 PMC9120705

[29] Coradduzza D, et al. Age-related cognitive decline, focus on microbiome: a systematic review and meta-analysis. Int J Mol Sci. 2023. 10.3390/ijms241813680. 37761988 10.3390/ijms241813680 PMC10531012

[30] Dong TS, Mayer E. Advances in brain–gut–microbiome interactions: a comprehensive update on signaling mechanisms, disorders, and therapeutic implications. Cell Mol Gastroenterol Hepatol. 2024;18(1):1–13. 38336171 10.1016/j.jcmgh.2024.01.024 PMC11126987

[31] Bremner JD, et al. Diet, stress and mental health. Nutrients. 2020. 10.3390/nu12082428. 32823562 10.3390/nu12082428 PMC7468813

[32] Zhao L, et al. Bidirectional gut-brain-microbiota axis as a potential link between inflammatory bowel disease and ischemic stroke. J Neuroinflammation. 2018;15(1):339. 30537997 10.1186/s12974-018-1382-3 PMC6290529

[33] Collins SM. Interrogating the gut-brain axis in the context of inflammatory bowel disease: a translational approach. Inflamm Bowel Dis. 2020;26(4):493–501. 31970390 10.1093/ibd/izaa004 PMC7054772

[34] Atanasova K, et al. Role of the gut microbiome in psychological symptoms associated with inflammatory bowel diseases. Semin Immunopathol. 2025;47(1):12. 39870972 10.1007/s00281-025-01036-x PMC11772462

[35] Piovani D, Armuzzi A, Bonovas S. Association of depression with incident inflammatory bowel diseases: a systematic review and meta-analysis. Inflamm Bowel Dis. 2023;30(4):573–84. 10.1093/ibd/izad109 PMC10988103 37300511

[36] Hu S, et al. Depression and anxiety disorders in patients with inflammatory bowel disease. Front Psychiatry. 2021;12:714057. 34690829 10.3389/fpsyt.2021.714057 PMC8531580

[37] World Health Organization. Mental health of older adults. 2023. Available from: https://www.who.int/news-room/fact-sheets/detail/mental-health-of-older-adults. Cited 19 Oct 2025.

