# Supplementary material for: Pooled prevalence of psychological distress and mental health conditions in older adults with inflammatory bowel disease: a protocol for a systematic review and meta-analysis
Source: Syst Rev. 2026 Feb 20;15:102. doi: 10.1186/s13643-026-03106-z (PMC13032432; doi:10.1186/s13643-026-03106-z)
Supplement: Supplementary file 1 — Supplementary Material 1 [file 13643_2026_3106_MOESM1_ESM.docx]

Appendix

MEDLINE

Ovid MEDLINE(R) ALL <1946 to January 14, 2026>

1 exp Inflammatory Bowel Diseases/ 109094

2 ("crohn* disease" or "crohn*" or "inflammatory bowel disease*" or "IBD" or "ulcerative colitis" or "colitis ulcerosa").tw,kf. 149587

3 1 or 2 163091

4 exp Aged/ 3816578

5 ("aged" or "older adults" or "older people" or "older persons" or "elderly" or "seniors" or "aging population" or "aged 60 and above" or "aged 60 years and older" or "age 60+" or "people over 60").tw,kf. 1307217

6 4 or 5 4550621

7 exp Mental Health/ 76500

8 ("mental health" or "mental wellbeing" or "mental well-being" or "mental stress*" or "mental disorder*" or "mental distress").tw,kf. 364062

9 exp Stress, Psychological/ 171935

10 (stress* or psychosocial or "psychosocial factor*" or "psychological distress" or "psychological factor*").tw,kf. 1488156

11 exp Depression/ 177629

12 depress*.tw,kf. 661112

13 exp Anxiety/ 132785

14 (anxiet* or anxious).tw,kf. 341784

15 exp mental disorders/ or exp mood disorders/ 1585138

16 ((mental* or psychiatric* or mood) adj3 (disorder* or illness* or condition*)).tw,kf. 236287

17 or/7-16 3578381

18 3 and 6 and 17 1104

19 limit 18 to yr="2014 -Current" 627

20 exp animals/ not humans.sh. 5414501

21 19 not 20 617

22 limit 21 to English language 610
