# Supplementary material for: Pooled prevalence of psychological distress and mental health conditions in older adults with inflammatory bowel disease: a protocol for a systematic review and meta-analysis
Source: Syst Rev. 2026 Feb 20;15:102. doi: 10.1186/s13643-026-03106-z (PMC13032432; doi:10.1186/s13643-026-03106-z)
Supplement: Supplementary file 2 — Supplementary Material 2 [file 13643_2026_3106_MOESM2_ESM.docx]

**Risk-of-Bias / Quality Assessment Templates**

Use these templates to assess risk of bias independently by two reviewers. Record a brief justification for each item. Resolve disagreements by consensus; if needed, adjudicate with a third reviewer.

**Table S1a**.  **Cross-sectional / Prevalence studies (JBI Checklist for Prevalence Studies)**

Outcome type: prevalence of depression, anxiety, and/or stress/psychological distress among older adults with IBD. Response options: Yes / No / Unclear / Not applicable.

Prevalence type (circle one): Point / Period / Unclear. Reference period/time window (if period): _____________.

| **Item** | **Signalling question** | **Judgement (Yes/No/Unclear/NA)** | **Justification/notes** | **Reviewer** |
| --- | --- | --- | --- | --- |
| 1 | Was the sample frame suitable for addressing the target population? |  |  | R1 / R2 |
| 2 | Were study participants sampled properly? |  |  | R1 / R2 |
| 3 | Was the sample size adequate? |  |  | R1 / R2 |
| 4 | Were the study subjects and the setting described in detail? |  |  | R1 / R2 |
| 5 | Was the data analysis conducted with sufficient coverage of the identified sample? |  |  | R1 / R2 |
| 6 | Were valid methods used for the identification of the condition (mental health outcome)? |  |  | R1 / R2 |
| 7 | Was the condition measured in a standard and reliable manner for all participants? |  |  | R1 / R2 |
| 8 | Was there an appropriate statistical analysis (for prevalence estimation)? |  |  | R1 / R2 |
| 9 | Was the response rate adequate, and if not, was the low response rate managed appropriately? |  |  | R1 / R2 |

Overall judgement (study-level): Low / Some concerns / High risk of bias (circle one). Justification:

**Table S1b. Cohort studies (Newcastle-Ottawa Scale - NOS)**

Use for longitudinal/comparative observational studies contributing to the association evidence stream (e.g., mental health predicting IBD outcomes or IBD predicting incident psychiatric disorders).

| **Domain** | **Item** | **NOS criterion** | **Star awarded? (Yes/No)** | **Justification/notes** | **Reviewer** |
| --- | --- | --- | --- | --- | --- |
| Selection | 1 | Representativeness of the exposed cohort |  |  | R1 / R2 |
| Selection | 2 | Selection of the non-exposed cohort (if applicable) |  |  | R1 / R2 |
| Selection | 3 | Ascertainment of exposure (IBD and/or mental health) |  |  | R1 / R2 |
| Selection | 4 | Demonstration that the outcome of interest was not present at the start of the study |  |  | R1 / R2 |
| Comparability | 5 | Comparability of cohorts based on the design or analysis (control for key confounders; at minimum age and sex, and where relevant IBD subtype and disease activity/severity or treatment intensity). Award up to 2 stars if the adjustment is clearly described. |  |  | R1 / R2 |
| Outcome | 6 | Assessment of outcome (IBD outcomes or incident psychiatric disorders) |  |  | R1 / R2 |
| Outcome | 7 | Was the follow-up long enough for outcomes to occur? |  |  | R1 / R2 |
| Outcome | 8 | Adequacy of follow-up of cohorts (loss to follow-up and handling) |  |  | R1 / R2 |

NOS cohort score: ____ / 9 stars. Overall judgement: Low / Moderate / High risk of bias (circle one).

**Table S1c. Case-control studies (Newcastle-Ottawa Scale - NOS)**

| **Domain** | **Item** | **NOS criterion** | **Star awarded? (Yes/No)** | **Justification/notes** | **Reviewer** |
| --- | --- | --- | --- | --- | --- |
| Selection | 1 | Is the case definition adequate? |  |  | R1 / R2 |
| Selection | 2 | Representativeness of the cases |  |  | R1 / R2 |
| Selection | 3 | Selection of controls |  |  | R1 / R2 |
| Selection | 4 | Definition of controls |  |  | R1 / R2 |
| Comparability | 5 | Comparability of cases and controls based on the design or analysis (control for key confounders; at minimum age and sex, and where relevant IBD subtype and disease activity/severity or treatment intensity). Award up to 2 stars if the matching/adjustment is clearly described. |  |  | R1 / R2 |
| Exposure | 6 | Ascertainment of exposure |  |  | R1 / R2 |
| Exposure | 7 | Same method of ascertainment for cases and controls |  |  | R1 / R2 |
| Exposure | 8 | Non-response rate |  |  | R1 / R2 |

NOS case-control score: ____ / 9 stars. Overall judgement: Low / Moderate / High risk of bias (circle one).

Note: For studies reporting both prevalence and associations, complete the JBI prevalence checklist for prevalence outcomes and the appropriate NOS checklist for association outcomes.

**Table S2a. Summary of Older Adults with IBD and Mental Health Conditions Across Included Studies**

One row per included study (or per older-adult subgroup if a study reports multiple older-adult cut-offs).

| **Study (Author, year)** | **Country/region** | **Design/setting/data source** | **Older-adult definition (≥60/≥65/other)** | **IBD subtype(s) included** | **Older-adult IBD sample (N)** | **Depression n/N (%)** | **Anxiety n/N (%)** | **Stress/psychological distress n/N (%)** | **MH ascertainment method/instrument/threshold** | **Prevalence type/timeframe (point/period; window if stated)** | **Notes on extractability (e.g., stratified only; derived; unclear)** |
| --- | --- | --- | --- | --- | --- | --- | --- | --- | --- | --- | --- |
|  |  |  |  |  |  |  |  |  |  |  |  |
|  |  |  |  |  |  |  |  |  |  |  |  |
|  |  |  |  |  |  |  |  |  |  |  |  |

**Table S2b. Stratified Summary by IBD Subtype and Mental Health Condition (older-adults subgroup)**

**Table 4b. Stratified (Long-Format) Summary by IBD Subtype and Mental Health Outcome**

One row per extractable estimate (e.g., subtype- and/or sex-stratified). This structure supports subgroup analyses without double-counting participants.

| **Study (Author, year)** | **Country/region** | **Design/setting/data source** | **Older-adult definition (≥60/≥65/other)** | **IBD subtype (CD / UC / IBD overall / IBD-U)** | **Sex stratum (overall/male/female)** | **MH outcome (depression/anxiety/stress)** | **Numerator (n)** | **Denominator (N)** | **Prevalence (%)** | **MH ascertainment method/instrument/threshold** | **Prevalence type/timeframe (point/period; window if stated)** | **Notes (e.g., subgroup only; overlapping reporting cautions)** |
| --- | --- | --- | --- | --- | --- | --- | --- | --- | --- | --- | --- | --- |
|  |  |  |  |  |  |  |  |  |  |  |  |  |
|  |  |  |  |  |  |  |  |  |  |  |  |  |
|  |  |  |  |  |  |  |  |  |  |  |  |  |
